# Paraneoplastic syndrome in undifferentiated embryonic sarcoma of the liver

**DOI:** 10.1186/s13550-020-0602-x

**Published:** 2020-02-18

**Authors:** Regine Mariette Perl, Almut Häring, Marius Stefan Horger, Christina Pfannenberg, Sergios Gatidis

**Affiliations:** 1 Department of Diagnostic and Interventional Radiology, University-Hospital of Tuebingen, Hoppe-Seyler-Straße 3, 72076 Tübingen, Germany; 2 Internal Medicine II, Department of Haematology, Oncology, Clinical Immunology and Rheumatology, University-Hospital of Tuebingen, Otfried-Müller-Straße 10, 72076 Tübingen, Germany

**Keywords:** Undifferentiated embryonic sarcoma of the liver (UESL), Paraneoplastic syndrome (PNS), Paraneoplastic leukemoid reaction (PLR), PET-CT, PET-MRI

## Abstract

**Background:**

The undifferentiated embryonic sarcoma of the liver (UESL) is a rare, aggressive tumor mainly affecting children. Since UESL has no specific clinical symptoms or imaging characteristics, many cases of UESL are diagnosed late. The paraneoplastic leukemoid reaction (PLR) is a very rare concomitant of oncological patients associated with poor prognosis. This report describes the clinical course of a patient combining these two rare entities and describes the diagnostic challenges and dynamics of paraneoplastic syndrome.

**Case presentation:**

We report a case of UESL in a 46-year-old male who became clinically conspicuous due to pronounced B symptoms. CT and MRI showed a suspicious unifocal liver lesion. As the histological analysis of a tissue sample did not reveal a clear result, an 18F-FDG-PET-CT examination was performed. In addition to a high glucose metabolism of the liver lesion, an increased glucose metabolism in the entire bone marrow was observed. This finding was considered as pronounced paraneoplasia and laparotomy with liver segment resection followed. Immediately after resection of the tumor the paraneoplastic symptoms completely declined and the patient showed no signs of recurrence in the 1-year follow-up.

**Conclusions:**

Although UESL is rare and predominantly affects children, this diagnosis should always be considered for unclear unifocal cystic liver lesions, regardless of the patient’s age, as appropriate treatment has a good prognosis.

## Introduction

Paraneoplastic syndromes are concomitants of cancer, which can occur in a variety of forms. Besides general manifestations such as fever, loss of body weight, and night sweats, special paraneoplastic symptoms may occur, which can be very specific in pointing to an underlying cancer. Identifying a patient’s symptoms as paraneoplastic allows for early diagnosis of cancer and can be used to monitor response to treatment or to detect recurrence [1, 2]. In this report, we describe the case of a 46-year-old patient with undifferentiated embryonic liver sarcoma (UESL). UESL is a rare tumor that predominantly occurs in children aged 6 to 10 years [3]. The incidence of this disease is very low at 0.6 to 1.2 cases per 1 million patients, and adults are affected by this tumor in only 10% of all cases [4, 5]. Originally, UESL was considered to be a very aggressive tumor with a poor prognosis, but due to advanced treatment options (advanced liver resection techniques and highly effective chemotherapeutic agents) prognosis has markedly improved [6].

## Case presentation

**Fig. 1:**
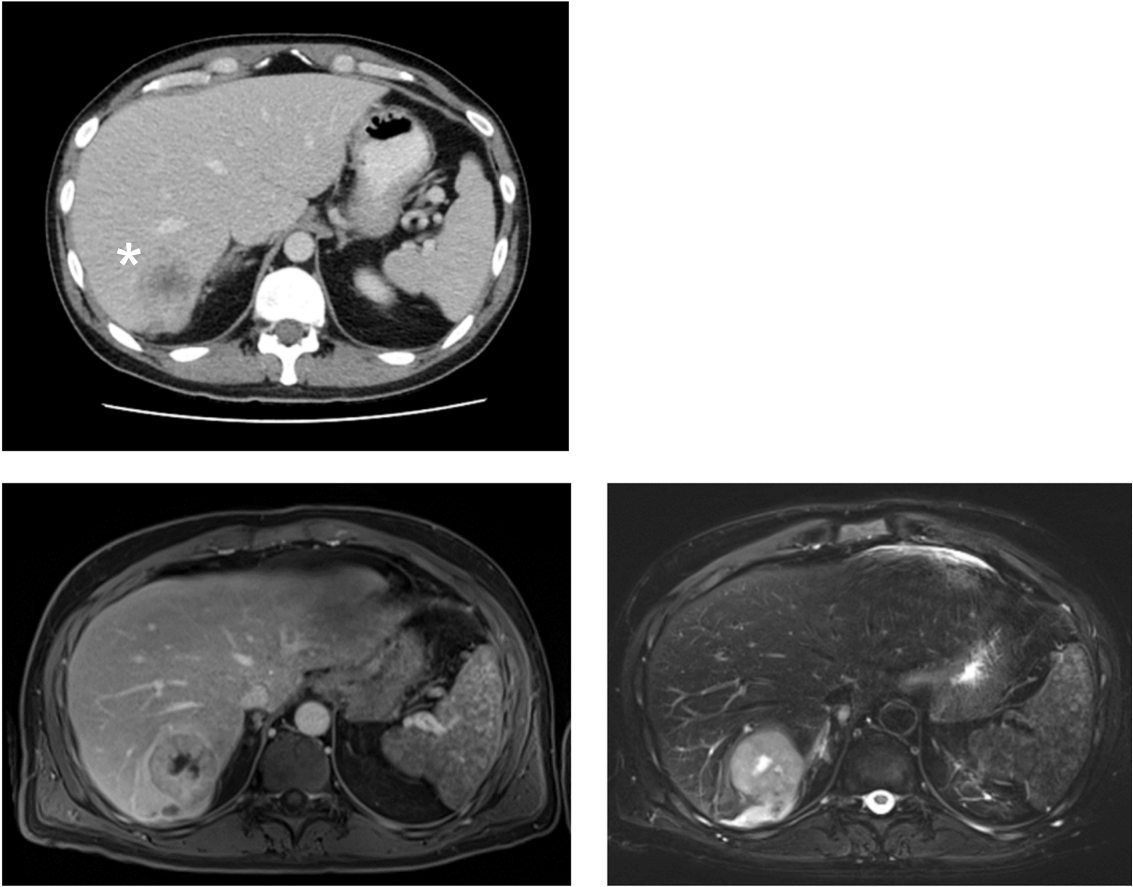
The initial CT abdomen during the portal venous phase in axial reconstruction (upper left) shows a hypodense lesion (*) in liver segment VII. In the MRI examination, the lesion had hypointense signal in the T1 vibe Dixon sequence (lower row on the left) and a hyperintensive signal in the T2 blade fs (bottom row on the right)

A 46-year-old male patient presented with weakness, lethargy, night sweats, unwanted weight loss (− 12 kg), and sudden onset of cough at a local physician. The physical examination was inconspicuous. Blood test showed increased inflammatory values (C-reactive protein (CRP) 6.2 mg/dl, normal < 0.5 mg/dl) and leukocytosis (leukocytes 21,900/μl), body temperature was initially not measured. After empirical antibiotic therapy had no effect, a CT scan was performed. Here, a lesion with an axial diameter of 4 cm was found in segment VII of the liver (Fig. 1). Extended laboratory tests 2 weeks later showed anemia (hemoglobin 11.3 g/dl), increased lactate dehydrogenase (278 U/l, normal ≤ 250), progredient CRP (18.27 mg/dl), and progressive leukocytosis (56.090/μl). The peripheral blood smear yielded 88.3% neutrophils, 4.3% lymphocytes, 4% monocytes, and < 1% eosinophils and basophils, respectively. Immature granulocytes were increased to 4.3% (norm ≤ 0.6%). Serum level of alfa-fetoprotein (AFP) was normal.

In the subsequent liver MRI examination, the lesion displayed an inhomogeneous T2-hyper and T1-hypointense signal (Fig. 1). In light of the clinical presentation, the lesion was initially regarded as a liver abscess and recommendation for histological assurance was given. The following sonographically controlled liver biopsy could not distinguish between undifferentiated sarcoma and poorly differentiated sarcoma, so the exact diagnosis remained unclear.

**Fig. 2:**
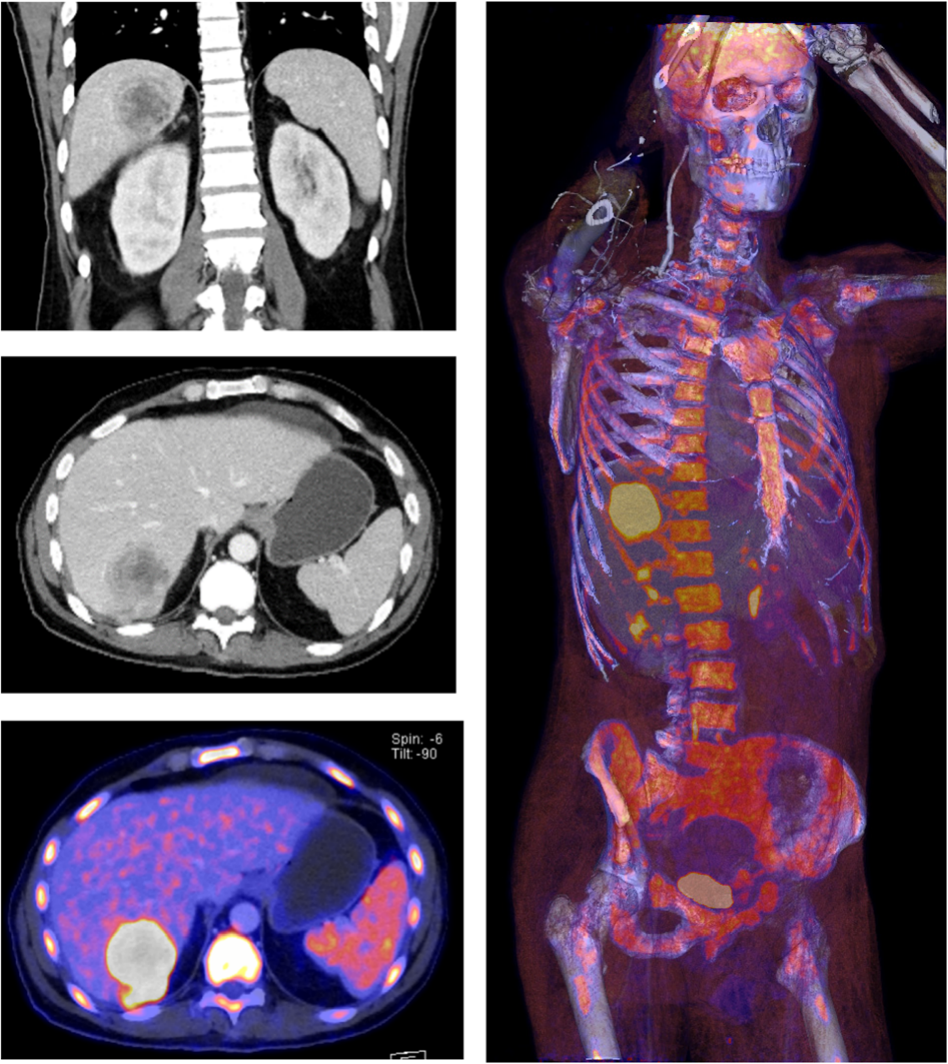
[^18^F] FDG whole-body PET-CT examination shows the known unifocal hypodense liver lesion and a distinct tracer uptake (SUV peak 33) without evidence of further suspicious lesions. Additionally, an increased signal in the entire bone marrow was found

**Fig. 3:**
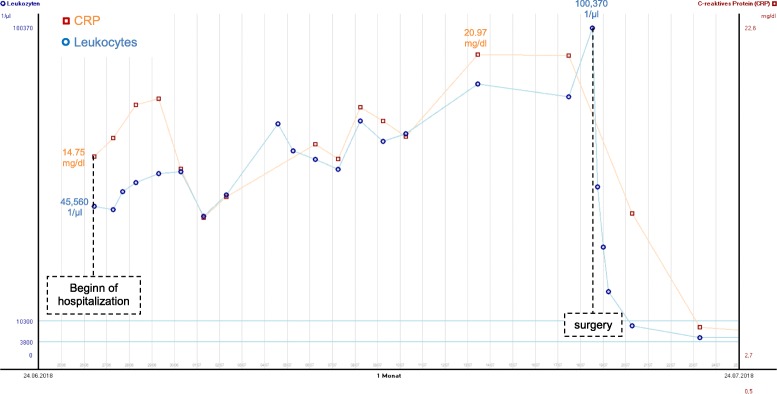
The inpatient admission of the patient was at a CRP of 14.75 mg/dl and increased leukocytes (45,560/μl). The CRP increased to a maximum of 20.97 mg/dl preoperatively, and the leukocytes reached their peak on the day of surgery with a value of 100,370/μl. Immediately postoperative both inflammatory values dropped sharply and leucocytes reached their normal values on the third postoperative day (8770/μl) and CRP on the 12th postoperative day (0.43 mg/dl)

To exclude further tumor manifestations, a whole-body [^18^F]-FDG PET-CT examination was performed 18 days after computed tomography. In this short-term interval, the lesion showed a slightly progressive diameter (5.3 × 6 cm vs. 4 cm) and a very high glucose metabolism activity (SUVpeak 33, SUVmax 41, SUVmean 23). PET-CT additionally showed significantly increased glucose metabolism in the entire bone marrow, which could be interpreted as unusually high bone marrow activation or as diffuse malignant bone marrow infiltration. There was no evidence of further suspicious lesions (Fig. 2). The following bone marrow puncture revealed a clearly hypercellular bone marrow with a pronounced increase in granulopoiesis and megakaryopoiesis and relatively reduced erythropoiesis. Although this finding was compatible with a myeloproliferative disease, it was considered pronounced paraneoplasia, which also explained the patient’s symptoms. Laparotomy with resection of liver segment VII followed. Immediately after surgery, the paraneoplastic symptoms of fever, cough, night sweats, high inflammatory values (CRP up to 20.9 mg/dl), and extreme leukocytosis (up to 100.370/μl) completely declined within hours (Fig. 3).

**Fig. 4:**
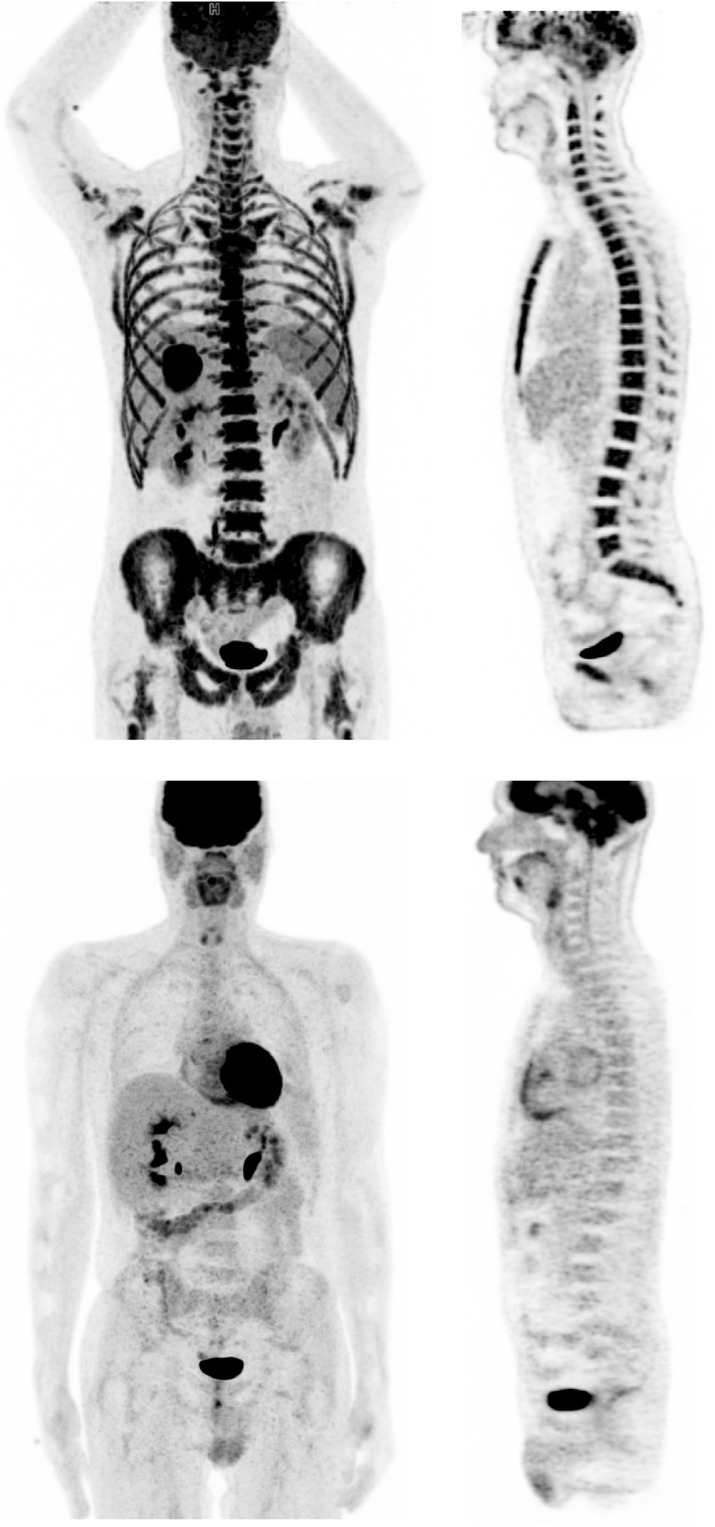
FDG PET tracer uptake before (upper row) and after (lower row) surgery: after resection of the tumor FDG PET showed complete normalization of bone marrow metabolism

Accordingly, in follow-up [^18^F]-FDG-PET-MRI, a complete normalization of bone marrow metabolism was observed (Fig. 4). Combination chemotherapy with actinomycin D, ifosfamide, and vincristine followed for 26 weeks. There was no proof of recurrence in the post-therapy CT and MRI examinations.

## Discussion

UESL is a rare mesenchymal tumor that predominantly occurs in children aged 6 to 10 years [3]. However, the literature also describes isolated cases in which adults were affected by this neoplasia. Patients usually experience non-specific gastrointestinal symptoms such as abdominal pain, nausea, and vomiting. In some cases, weight loss as well as a palpable tumor in the upper abdomen are described [4–6]. In addition, the radiological properties of a UESL are rather unspecific. Computed tomography typically reveals a well-defined, unifocal hepatic lesion with a cystic component that represents areas of necrosis and hemorrhages [7, 8]. In MRI studies, UESL are predominantly described as inhomogeneous T1-hypo- and T2-hyperintensity lesions with cystic parts [4, 7]. The interaction of missing clinical symptoms and unspecific radiological characteristics contributes to the fact that most patients are diagnosed late so the tumors are already very large at the time of diagnosis. Gabor et al. [7] described a case series of 15 patients with UESL with a mean tumor diameter of 13.5 cm and rapid growth in 2 of these patients. In our case, the tumor showed a size increase of approx. 1.3 cm within 18 days, so that here, too, rapid tumor growth can be assumed. Some case reports describe tumors measuring up to 25 × 19 × 14 cm [5], and there are case reports in which the patients became clinically conspicuous only after the tumor had ruptured [9–11]. Due to the pronounced paraneoplastic syndrome in this case, the patient became clinically noticeable relatively early, so that the diagnosis could be made at a comparatively small tumor diameter of 5.3 × 6 cm.

The term paraneoplastic syndrome (PNS) refers to the symptoms of patients with malignant neoplasms that are due to damage to organs or tissues at sites remote from neoplasms [12]. They can affect various organ systems, including the hematological system [2]. In our case, in addition to typical B symptoms (fatigue, night sweats, weight loss), the patient showed pronounced leukocytosis up to 100,370/μl and increase in immature granulocytes (4.3%; norm ≤ 0.6%). Reactive leukocytosis with a white blood cell count exceeding 50,000/μl and increased neutrophilic precursors is defined as leukemoid reaction (LR) [13], so that in the present case a paraneoplastic leukemoid reaction can be presumed. Interestingly, all paraneoplastic symptoms as well as the conspicuous laboratory parameters normalized immediately after resection of the UESL. There is another case report in the literature of a 9-year-old girl with UESL in which tumor resection also resulted in the rapid resolution of the PNS. In her case, however, the PNS consisted of a refractory long QTc syndrome, torsade de pointe, fever of unknown origin, and secretory diarrhea [12].

Extreme leukocytosis in the form of a paraneoplastic leukemoid reaction (PLR) is a very rare disease. Granger et al. report that of 758 patients with solid tumors and extreme leukocytosis, only 77 (10%) had PLR. According to Granger, the prognosis of patients with PLR is poor and 78% of patients died within 12 weeks after the first evidence of hyperleukocytosis. Only 10% of patients survived the malignancy for more than 1 year after successful treatment of the underlying malignancy [14]. The patient we report on is, 1 year after therapy, still alive and showed no signs of recurrence in the follow-up examinations.

## Conclusion

The present case is remarkable in two respects: first, the diagnosis of UESL in adults is a rarity; second, it describes the diagnostic challenges and the dynamic of PNS in an impressive way. Since UESL does not have specific clinical symptoms nor characteristic imaging features that allow for unambiguous assignment, the diagnosis of this malignancy remains a challenge. Even if UESL is rare and predominantly found in children, this diagnosis should always be considered regardless of the patient’s age in case of unclear unifocal cystic liver lesions as an adequate treatment by now has a good prognosis.

## Data Availability

All relevant data is included in the manuscript.
